# New earwigs in mid-Cretaceous amber from Myanmar (Dermaptera, Neodermaptera)

**DOI:** 10.3897/zookeys.130.1293

**Published:** 2011-09-24

**Authors:** Michael S. Engel

**Affiliations:** 1 Division of Entomology (Paleoentomology), Natural History Museum and Department of Ecology & Evolutionary Biology, 1501 Crestline Drive – Suite 140, University of Kansas, Lawrence, KS 66049-2811, USA; 2 Division of Invertebrate Zoology, American Museum of Natural History, Central Park West at 79th Street, New York, NY 10024-5192, USA

**Keywords:** Albian, Amber, Mesozoic, Earwigs, Polyneoptera, Pygidicranidae, Diplatyidae

## Abstract

Two new earwigs (Dermaptera) recently discovered in mid-Cretaceous (latest Albian) amber from Myanmar are described and figured. *Astreptolabis ethirosomatia* **gen. et sp. n.** is represented by a peculiar pygidicranoid female, assigned to a new subfamily, Astreptolabidinae **subfam. n.**, and differs from other protodermapterans in the structure of the head, pronotum, tegmina, and cercal forceps. *Tytthodiplatys mecynocercus* **gen. et sp. n.** is a distinctive form of first-instar nymph of the Diplatyidae, the earliest record for this basal earwig family. The taxon can be distinguished from other Early Cretaceous nymphs by the structure of the head, antennae, legs, and most notably its filamentous and annulate cerci. The character affinities of these taxa among Neodermaptera are generally discussed as is the identity of an enigmatic ‘earwig-like’ species from the Jurassic of China.

## Dedication

It is with great pleasure that I dedicate this brief contribution to my friend and colleague, Dr. Alexandr P. Rasnitsyn, one of the great statesmen of paleoentomology. For the last 52 years Alex has produced some of the most influential works in the field, fueling the interests and investigations of generations of subsequent students of both Hymenoptera and fossil insects in general. On the same day Alex marks his 75th year, I shall mark my 40th. If in the coming 35 years I can undertake merely a similar fraction of what he has achieved, then I shall consider myself pleased. It is with considerable pride that Alex may look back on a career of tremendous accomplishment, and we all look forward to many more years of such successful endeavors from him.

## Introduction

Earwigs are certainly one of the lesser-studied lineages of insects, with comparatively few current investigations underway into their diversity, behavior, biology, and general natural history. This is unfortunate given the remarkable diversity of form for these often subsocial insects, with their prominent and immediately recognizeable cercal forceps which are used in aggressive/defensive interactions, courtship, and prey capture (e.g., Günther and Herter 1974; Briceño and Eberhard 1995; Haas 2003; Costa 2006; Rankin and Palmer 2009). The systematics of the group was once the concerted interest of dermapterological luminaries such as Malcolm Burr (1876–1954), Walter D. Hincks (1906–1961), and Allan Brindle (1915–2001) but has not received quite as much effort in recent years and the sizeable monographs that were once regularly flowing from earwig taxonomists has slowed. Ironically, paleontological investigation into earwigs has seen a reversed trend, with increasingly more and more accounts during the last decade (e.g., Nel et al. 2003; Wappler et al. 2005; Chatzimanolis and Engel 2010; and additional citations below). Of particular interest have been the numerous new records of Mesozoic Dermaptera which have come to light, mostly as compression fossils from Asia (e.g., Zhang 1997; Engel et al. 2002; Zhang 2002; Zhao et al. 2010a, 2010b, 2011) or South America (Engel and Chatzimanolis 2005; Haas 2007), but also including a steadily accumulating number of amber inclusions (e.g., Engel and Grimaldi 2004; Engel 2009; Perrichot et al. 2011; Engel et al. 2011).

Herein I provide the description of two newly recognized earwigs in mid-Cretaceous amber from Myanmar. The morphology and possible affinities of these taxa are discussed as is the attribution to Dermaptera of some recently described enigmatic insects from the Jurassic of China (Zhao et al. 2010a), and which have some superficial similarities to one of the species considered here.

## Material and methods

The material discussed herein originates from the latest Albian amber deposits of northern Myanmar in the State of Kachin. The general paleobiota, dating, and origin of this amber have been overviewed by Zherikhin and Ross (2000), Grimaldi et al. (2002), Cruickshank and Ko (2003), and Ross et al. (2010). All material is deposited in the Amber Fossil Collection, Division of Invertebrate Zoology (Entomology), American Museum of Natural History, New York. The classification followed herein is that of Engel and Haas (2007), while the morphological terminology and format for the descriptions generally follow those of Giles (1963), Günther and Herter (1974), Haas (1995), Engel et al. (2011), and Perrichot et al. (2011).

## Systematic Paleontology

### Order Dermaptera De Geer

**Suborder Neodermaptera Engel**

**Family Pygidicranidae Verhoeff**

#### Astreptolabidinae

Engel subfam. n.

urn:lsid:zoobank.org:act:FF43BCB1-914D-48DC-9EE6-BA1331525633

http://species-id.net/wiki/Astreptolabidinae

##### Type genus.

*Astreptolabis* Engel, gen. n.

##### Diagnosis.

**Figure 1.**
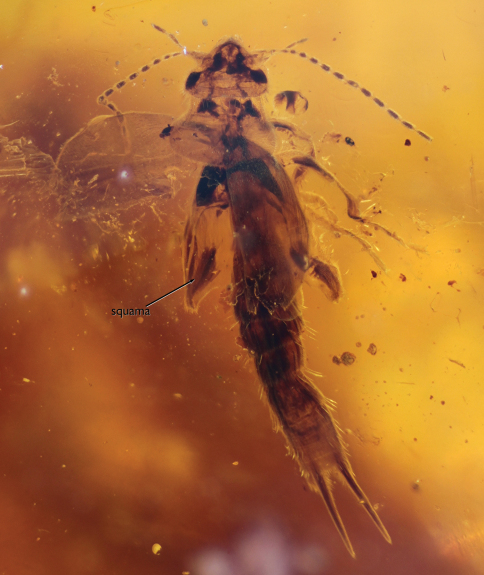
Dorsal aspect photomicrograph of holotype female of *Astreptolabis ethirosomatia* gen. et sp. n. (AMNH Bu-FB20). In this orientation the head is slightly dipped forward making the postocular area appear minutely foreshortened relative to the compound eyes.

Female: Minute earwigs (ca. 3.5 mm in length); somewhat dorsoventrally compressed; densely setose, but not chaetulose; integument dull and matt. Head prognathous, broad, slightly broader than anterior border of pronotum (Fig. 1), apparently tumid, posterolateral corners gently curved, posterior border straight; compound eyes well developed, prominent, separated from posterior border of head by slightly less than compound eye length, setose; ocelli absent; antenna with at least 14 antennomeres (an unusually small number for basal Neodermaptera and likely autapomorphic for this subfamily), scape stout, pedicel longer than wide, flagellomeres longer than wide, progressively more elongate from flagellomere II–X, with X–IV subequal in size. Pronotum exceptionally large (Fig. 1), anterior margin relatively straight, posterior border gently convex, lateral borders slightly divergent in anterior half, flared and convex in posterior half, posteriorly broader than head, all borders ecarinate. Tegmina present, without venation, symmetrical, elongate, outer margins convex, apex gently curved and tapering to midline (not truncate), covering first four abdominal segments (Fig. 1); hind wings present, with squama slightly exposed from under tegmina. Femora apparently not carinulate; tarsi trimerous, second tarsomere shortest, not extending beneath base of third tarsomere; pretarsal ungues simple; arolium absent. Abdomen slender, elongate (eight visible segments, typical for females), lateral margins parallel-sided, most segments only slightly wider than long, apicalmost segment with straight apical margin, without tubercles. Cerci symmetrical, slightly longer than apicalmost three abdominal segments, straight, tubular, gently tapering to acute apex, densely covered in microtrichia, without tubercles, dentition, or serrations, broadly separated at base (Fig. 1); pygidium not evident; valvulae not exposed at abdominal apex.

Male: Unknown.

#### Astreptolabis

Engel gen. n.

urn:lsid:zoobank.org:act:D1E57007-13A3-45BD-B732-D77235E57FF8

http://species-id.net/wiki/Astreptolabis

##### Type species.

*Astreptolabis ethirosomatia* Engel, sp. n.

##### Diagnosis.

As for the subfamily (*vide supra*).

##### Etymology.

The new genus-group name is a combination of the Greek words *astreptos* (meaning, “not curved”) and *labis* (meaning, “forceps”). The name is feminine.

#### Astreptolabis ethirosomatia

Engel sp. n.

urn:lsid:zoobank.org:act:EEF799CF-0A18-420D-B228-58E3CC0851E0

http://species-id.net/wiki/Astreptolabis_ethirosomatia

Fig. 1

##### Holotype.

AMNH Bu-FB20; adult female; amber, mid-Cretaceous, Myanmar: Kachin State (nr. Myitkyina), ex coll. Federico Berlöcher; deposited in the Division of Invertebrate Zoology (Entomology), American Museum of Natural History, New York.

##### Diagnosis.

As for the genus (*vide supra*).

##### Description.

As for the subfamily and genus, with the following additions: Female: Total length as preserved (including cerci) ca. 3.5 mm; head medial length from clypeal apex to posterior border 0.38 mm, maximum width (across level of compound eyes) 0.56 mm; compound eye length 0.13 mm; length of head behind compound eye 0.11 mm. Pronotum medial length 0.45 mm, anterior width 0.47 mm, posterior width 0.70 mm; tegmen length 1.21 mm, maximum width 0.49 mm. Abdominal length as preserved (excluding cerci) 1.65 mm, maximum width 0.44 mm; cercal forceps length 0.65 mm, basal width 0.05 mm, separation between bases 0.12 mm. Integument as preserved apparently brown to dark brown, impunctate, dull, matt throughout. Legs without spines or bristle-like setae. Setae of body short and dense except more elongate setae posterolaterally on abdominal terga (Fig. 1).

Male: Unknown.

##### Etymology.

The specific epithet is a combination of the Greek words *etheira* (meaning, “hairy”) and *somation* (diminutive form of the word for, “body”).

### Family Diplatyidae Verhoeff

#### Tytthodiplatys

Engel gen. n.

urn:lsid:zoobank.org:act:F81ABA76-CE78-4616-BE31-01FB81CE0DDE

http://species-id.net/wiki/Tytthodiplatys

##### Type species.

*Tytthodiplatys mecynocercus* Engel, sp. n.

##### Diagnosis.

**Figure 2.**
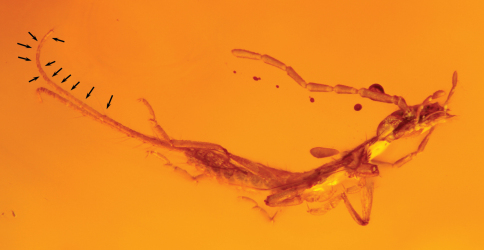
Photomicrograph of holotype nymph of *Tytthodiplatys mecynocercus* gen. et sp. n. (AMNH Bu-FB75) (arrows indicate most easily discernible cercomere joints).

Minute earwigs (ca. 1.9 mm in length excluding cerci), with eight antennomeres (groundplan condition for first instars of Neodermaptera). Body dorsoventrally compressed (Fig. 2), with sparsely scattered setae, not chaetulose; integument dull and matt. Head prognathous, slightly broader than long (estimated as direct dorsal view of specimen not possible: Fig. 2), somewhat tumid, posterior angles rounded, posterior border relatively straight, rounded (not truncate or concave); compound eyes well developed, somewhat prominent, separated from posterior border of head by slightly more than compound eye diameter; ocelli absent; antenna with eight articles, scape relatively slender, pedicel short, subquadrate, very slightly wider than long, meriston longer than other flagellomeres; mouthparts typical for Dermaptera (e.g., Waller et al. 1996). Pronotum and mesonotum roughly subquadrate, slightly narrower than head, with anterior and posterior angles acutely rounded, lateral borders weakly convex, all borders ecarinate; pronotal median longitudinal furrow (= sutura pronotalis longitudinalis) not evident; metanotum broader than maximum length, anterior border straight, lateral borders ecarinate and diverging posteriorly, posterior border broadly concave. Legs not greatly elongate; procoxae apparently near posterior border of prosternum; femora not carinulate or compressed; tibiae relatively short, about as long as tarsi; tarsi trimerous, second tarsomere greatly shortened, not widened apically, scarcely extending apically beneath third tarsomere; pretarsal ungues simple, arolium absent. Abdominal terga sculptured as on thoracic nota; segments transverse, apicalmost segment much smaller than penultimate segment; cerci greatly elongate, about as long as combined lengths of abdomen and thorax, filamentous, annulated (as in nymphs of Diplatyidae and Karschiellidae) (Fig. 2), with bases broadly separated.

##### Etymology.

The new genus-group name is a combination of the Greek word *tytthos*, meaning “small” or “young”, and *Diplatys*, type genus of the family (itself a combination of the Greek words *di* and *platys*, meaning “two” and “broad”, respectively). The name is masculine.

#### Tytthodiplatys mecynocercus

Engel sp. n.

urn:lsid:zoobank.org:act:C49383C4-8C91-49BE-ADDA-06B45D854AAE

http://species-id.net/wiki/Tytthodiplatys_mecynocercus

Fig. 2

##### Holotype.

AMNH Bu-FB75; female nymph (first instar); amber, mid-Cretaceous, Myanmar: Kachin State (nr. Myitkyina), ex coll. Federico Berlöcher; deposited in the Division of Invertebrate Zoology (Entomology), American Museum of Natural History, New York.

##### Diagnosis.

As for the genus (*vide supra*).

##### Description.

As for the genus with the following minor additions: First-instar nymph: Total length as preserved (including cerci) ca. 1.9 mm; head medial length from clypeal apex to posterior border 0.46 mm; compound eye length 0.08 mm; length of head behind compound eye 0.10 mm. Pronotum medial length 0.25 mm; mesonotum medial length 0.22 mm; metanotum medial length 0.12 mm. Abdominal length as preserved (excluding cerci) 0.83 mm; cerci length 1.58 mm. Integument as preserved apparently brown, impunctate, strongly imbricate, dull, matt throughout. Legs without spines or bristle-like setae except for a few stiff setae on dorsal surface of profemora. Cerci separated at base by about cercal basal width. Setae of body sparse, where present setae stiff and erect to suberect, particularly those apicolaterally on abdominal terga; cerci with numerous, elongate, stiff, erect setae scattered along cercomeres.

##### Etymology.

The specific epithet is a combination of the Greek words *mekyno* (meaning, “prolong”) and *kerkos* (meaning, “tail”).

## Discussion

Discovery of these two specimens brings the diversity of earwigs in Burmese amber to four species – *Myrrholabia electrina* (Cockerell 1920), *Burmapygia resinata* Engel and Grimaldi (2004), *Atreptolabis ethirosomatia* gen. et sp. n., and *Tytthodiplatys mecynocercus* gen. et sp. n., none of which are of the Eudermaptera furthering the notion that Eudermaptera are of later Cretaceous or Early Tertiary origin. This is also true if we look at other Cretaceous amber earwigs, namely *Rhadinolabis phoenicica* Engel et al. and an unnamed nymph in Lebanese amber (Engel et al. 2011), and the unnamed nymphs and *Gallinympha walleri* Perrichot and Engel in French amber (Engel 2009; Perrichot et al. 2011). There are earwigs in New Jersey amber but these are too poorly preserved to permit conclusive assignment beyond Neodermaptera (Engel pers. obs.) and it is hoped that better material eventually shall be discovered.

*Astreptolabis* is easily placed among the Neodermaptera owing to the absence of ocelli, the trimerous tarsi, unsegmented cerci, vestigial ovipositor, and absence of venation in the tegmina (Willmann 1990; Haas and Klass 2003; Grimaldi and Engel 2005). The mouthparts with small mandibles concealed under the labrum and the galea and lacinia prominent are also of typical dermapteran form (Giles 1963; Waller et al. 1996). The presence of only eight visible abdominal tergites is indicative of a female. While the cerci are immediately distinctive for this female, males are likely to have had significantly different cercal forms (as is typically the case between males and females of the same species among living Dermaptera). As such, males of *Astreptolabis ethirosomatia* would not be recognized on the basis of similarly straight, tapering, and tubular cerci but more likely have similar setation, integumental sculpturing, and head, pronotal, tarsal, and tegminal structure. The dense setation of the body, small size and dorsoventrally compressed body, broad head, symmetrical cercal forceps, and the non-transverse antennomeres IV–VI are all indicative of a pygidicranid among primitive neodermapteran families. Unfortunately, the arrangement and structure of the ventral cervical sclerites cannot be discerned in the holotype. Interestingly, there is some superficial similarity between *Astreptolabis* and the epizoic Hemimeridae most prominently the straight, broadly separated and setose cerci, exceptionally broad head, large pronotum, and posterolateral patches of elongate setae on the abdominal terga (e.g., Giles 1963; Günther and Herter 1974; Nakata and Maa 1974). However, each of these characters is variable enough across dermapteran families such that they cannot be considered synapomorphic and these taxa differ in innumerable other characters such as the absence of tegmina and wings, absence of eyes, shape of the abdomen, and the strikingly modified legs and tarsi in hemimerids.

Astreptolabidinae can be readily differentiated from other pygidicranid subfamilies by the peculiar form of the female cercal forceps, as well as the combination of a broad, somewhat truncate head, large pronotum, and densely and finely setose body. From Burmapygiinae, also in Burmese amber, the new subfamily further differs in the stout scape, the absence of the arolium, and the shorter valvulae which do not apparently extend beyond the apex of the subgenital plate. In some respects, the head and pronotal structure of *Astreptolabis* are reminiscent of Echinosomatinae (head broad, transverse, somewhat truncate posteriorly; pronotum large, subquadrate or transverse; scape stout), but the absence of chaetae on the integument (instead dense, fine setae), particularly the head, pronotum, and tegmina, excludes inclusion therein. Echinosomatines are also rather large, broad, and stout (ca. 8–30 mm in length), never as minute as Astreptolabidinae, and although the cercal forceps are simple (i.e., lacking dentition or serrations) and generally widely separated as in the latter, they are distinctly arcuate and more stout in the former. In addition, the female valvulae of echinosomatines typically extend slightly beyond the apex of the subgenital plate. Astreptolabidinae may represent an early ally of the Echinosomatinae.

It is interesting to note that the tegminal form and minute body size are reminiscent of the recently described *Atopderma ellipta* Zhao et al. (2010a) from the Jurassic-age Daohugou deposits of Inner Mongolia (a junior synonym of *Leicarabus parvus* Hong), although the abdominal form (and thusly the cercal or other terminal structures) remains unknown for this enigmatic insect (although it may be misplaced in Dermaptera, *vide infra*). From the superficial shape of the tegmina (note that these may be elytra in *Astreptolabis ellipta*, *vide infra*) alone it is tantalizing to speculate that these species might be related. However, most observable features are not shared between the two taxa as *Astreptolabis* was clearly very setose while *Astreptolabis ellipta* apparently was not, and more importantly the overall morphology of the head, pronotum, and tarsi are dramatically different. Indeed, there is reason to question the assignment of *Astreptolabis ellipta* to Dermaptera at all, and certainly within Neodermaptera, a placement considered likely by the original authors (Zhao et al. 2010a). Unlike all Neodermaptera, *Astreptolabis ellipta* apparently had pentamerous tarsi (or at least more tarsomeres than the trimerous condition of all living and fossil Neodermaptera). Similarly non-dermapteran in character is the assertion that the tarsus of *Astreptolabis ellipta* is as long as the tibia, a feature more similar to that among some Staphyliniformia than many adult, non-epizoic Dermaptera (although the condition certainly can be found). The statement that the “ventral cervical sclerites of equal size” (p. 463) does not match with the condition for *Astreptolabis ellipta* is enigmatic in that these structures are neither described nor figured in the account of the genus or species, and from the photographs of the specimens would not be discernible in the series either (these are often difficult to discern in living and amber-preserved specimens and their recognition on a few-millimeter-long compression fossil is unlikely). In Diplatyidae, Karschiellidae, and Pygidicranidae the ventral cervical sclerites are of equal size, with the posterior sclerite well separated or only very medially bordering the prosternum (i.e., the ‘blattoid’ neck of Popham 1959, 1965, 1985). Zhao et al. (2010a) pinned their attribution onto five features which they considered conclusively earwig in form. I must respectfully disagree with my colleagues here as I see nothing supporting placement in Dermaptera among their arguments. To be more precise (taking their points in the order with which they were presented):

1. Head shape: The assertion that in Staphyliniformia the head is much narrower than the pronotum is incorrect, and triangular heads are very common in Staphylinidae (as well as numerous other beetle families). Yes, there are many staphylinids in which the pronotum is narrower than the head, but there are similarly many in which the head is as broad as or broader than the pronotum (e.g., many Oxytelinae, Staphylininae, Scydmaeninae, &c.), not to mention this similar condition among other families such as Hydraenidae, Hydrophilidae, &c. The head shape of *Astreptolabis ellipta* certainly does not exclude placement in Coleoptera. Moreover, the total antennal number (unknown for the holotype and unreported for the paratypes which have more completely preserved antennae), seems to be approximately 11 in some specimens (momentarily overlooking the concern that some of the paratypes do not appear to be conspecific with the holotype; some of the paratypes look more dermapteran-like while the holotype looks awfully coleopteran), a distinctly polyphagan, if not also staphyliniform, groundplan condition. What is known of the head and antenna in *Astreptolabis ellipta* neither supports nor refutes a placement in Coleoptera or Dermaptera.

2. Prothorax without pleural sulcus separating pronotum and propleuron: This is an enigmatic character for ‘distinguishing’ between these orders, and it is not entirely clear what difference the authors are really referring to as both Staphyliniformia and Dermaptera have a sulcal separation between the pronotum and propleuron (e.g., Blackwelder 1936; Giles 1963; Günther and Herter 1974; Naomi 1988; Waller et al. 1999). As briefly described by Zhao et al. (2010a) this does not separate Coleoptera from Dermaptera, and it is unlikely that such a feature would have been visible based on the orientation of the available specimens.

3. ‘Tegmina’ purportedly long and thin, and with curved outer (costal) margins: Certainly most staphylinids do have short, more rectangular elytra, but many Staphyliniformia may also have more elongate elytra, and definitely of the form appearing in *Astreptolabis ellipta*. For example, some Scydmaeninae, Omaliinae, and Scaphidiinae have more fully-developed elytra, particularly of the latter subfamily. Lastly, the assertion that Coleoptera should have more heavily sclerotized elytra than the condition observed in *Astreptolabis ellipta* is both *ad hoc* and erroneous. No one could argue that the reduced elytra of many Ripiphoridae, or those of Meloidae, Melyridae, Phengodidae, Pyrochroidae, or even some Coccinellidae are more heavily sclerotized than those of *Astreptolabis ellipta*. There is certainly a considerable range of cuticle thickness and development across beetle elytra (e.g., Kamp and Greven 2010). This is not to say *Astreptolabis ellipta* belongs to any of these families (not by any stretch of the imagination), but simply serves to demonstrate that the *ad hoc* assertion that the elytra of *Astreptolabis ellipta* are not strong enough to be coleopteran is not factually based.

4. Carinated and spined femora: Although the authors of *Astreptolabis ellipta* state that it has carinulate femora, this is not the case in any of their figures. Some primitive earwigs have femora with visible lamelliform edges dorsally but the presumed carina referred to by Zhao et al. (2010a) appears to be a ventral ridge demarcating the under surface of the profemur, a feature common of many insect femora. I believe the authors have misinterpreted the condition in earwigs. Similarly, none of their figures show spines on the femora, nor do the descriptions mention any such features, and so it is unclear what Zhao et al. (2010a) are referring to. Regardless, femoral spines are not diagnostic of Dermaptera and many earwigs lack such structures. In addition, the close position of the procoxae in *Astreptolabis ellipta* (Zhao et al. 2010a) is much more like a coleopteran (e.g., Staphylinidae) than an earwig in which the procoxae are almost always separated by a sizeable portion of the prosternum (e.g., Giles 1963; Waller et al. 1999).

5. Abdominal shape: That the abdominal terga in Dermaptera are always transverse and the implicit assumption from the account of Zhao et al. (2011) that parallel lateral abdominal margins is indicative of Staphylinidae and not Dermaptera is simply false. The form of the terga is quite variable across Dermaptera, many are truly transverse, but some can be nearly quadrate and elongate in form. Similarly, the lateral margins of the abdomen can be convex or parallel-sided, particularly so among the more proximal segments (e.g., Hincks 1955; Günther and Herter 1974; Steinmann 1986). Lastly, it is presumably the presence of laterotergites that Zhao et al. (2010a) are referring to when they mention that the “tergum is slightly narrower than the sternum” in staphylinids, with the laterotergites being confused for upturned sternal margins. Regardless, the described condition is not universally true for Staphylinidae, and most certainly not the case for Staphyliniformia (e.g., Blackwelder 1936; Naomi 1989). Moreover, the abdomen is not preserved in the holotype of *Astreptolabis ellipta* and at most by 2–3 segments in some of the paratypes. From these basal portions alone nothing can be derived that is strictly dermapteran in character, although these segments are not necessarily ‘staphylinid-like’ either.

The purpose here is not to assert that *Astreptolabis ellipta* is a beetle, and more precisely a staphyliniform beetle, but to demonstrate instead that a coleopteran attribution cannot be so readily dismissed by the stated characters. Naturally, dramatically autapomorphic taxa can appear in any period of time and such may be the case with *Astreptolabis ellipta* should more complete material reveal definitive dermapteran synapomorphies. Thorough redescriptions of the type series are needed but more critical will be the discovery of more complete and finely-preserved specimens. Only then will accurate conclusions on the phylogenetic affinities of these wonderful animals be permitted. For the time being, however, I conservatively consider *Astreptolabis ellipta* to be of uncertain ordinal assignment.

On the surface the phylogenetic affinities of *Tytthodiplatys mecynocercus* would appear more challenging than those of *Astreptolabis* given that the morphology and systematics of immature Dermaptera has not received as much attention as that of the adults. Indeed, many challenges remain for making conclusive statements about fossil earwig nymphs (e.g., Engel 2009; Engel et al. 2011; Perrichot et al. 2011). Some of the most important works are Günther and Herter (1974), Brindle (1987), and Matzke and Klass (2005), but concentrated efforts are still needed in the study of nymphal Dermaptera. Nonetheless, among all of the fossil nymphs *Tytthodiplatys mecynocercus* is perhaps the easiest to assign to family. Firstly, the epizoic families Hemimeridae and Arixeniidae can be excluded on the basis of their numerous peculiar modifications associated with their life histories (e.g., Giles 1961, 1963; Davies 1966; Günther and Herter 1974; Nakata and Maa 1974; Waller et al. 1999; Klass 2001), leaving only the non-parasitic families of the Neodermaptera. The elongate, filamentous and annulate cerci are a plesiomorphic trait known only in the basal families Diplatyidae and Karschiellidae. In these families the adult cercal forceps develop from the basalmost cercomere of the nymph (Green 1896, 1898) and it is likely that the loss of annulations (resulting from the loss of all but the basalmost cercomere) represents a synapomorphy for Neodermaptera exclusive of Karschiellidae and Diplatyidae (as clade Cercodermaptera). The annulations are difficult to discern in the holotype but are most easily observed toward the upturned apical portion of the right cercus, where at least four distinct subunits are observable. The lack of carinulate femora and the large compound eyes are indicative of a diplatyid rather than a karschiellid. Accordingly, *Tytthodiplatys mecynocercus* is considered the earliest representative of the ancient earwig family Diplatyidae. Staging the nymph as a first instar is based on the exceptionally small size coupled with the undeveloped antenna with only eight antennomeres, the condition typical to first-instar nymphs in Neodermaptera (where known). The count of eight antennomeres only extends into the second instar among the Eudermaptera, seemingly reflective of the generally lower number of adult antennomeres in this clade relative to other neodermapteran families (e.g., Matzke and Klass 2005).

The Cretaceous amber record of Dermaptera is steadily growing and it is only a matter of time before informative new specimens are discovered in the deposits of Spain, Canada, and elsewhere (e.g., Peñalver and Delclòs 2010; McKellar and Wolfe 2010), alongside new material from Tertiary sources such as Mexico, India, or Australia (e.g., Solórzano-Kraemer 2007, 2010; Rust et al. 2010; Hand et al. 2010). As the wealth of available material continues to grow it is likely that the amber record for earwigs will become as informative for dermapteran phylogeny as have the treasures of fossil ants, bees, and termites that have amassed during the last 15 years for understanding their respective genealogies (e.g., Grimaldi et al. 1997; Grimaldi and Agosti 2000; Engel 2001, 2011; Grimaldi and Engel 2005; Perrichot et al. 2008a, 2008b; Grimaldi et al. 2008; Engel et al. 2007, 2009). As has been the case for these other lineages, our knowledge of Mesozoic earwigs has shifted considerably in these same 15 years and will undoubtedly continue to do so. Remarkable discoveries certainly await us.

## Supplementary Material

XML Treatment for Astreptolabidinae

XML Treatment for Astreptolabis

XML Treatment for Astreptolabis ethirosomatia

XML Treatment for Tytthodiplatys

XML Treatment for Tytthodiplatys mecynocercus

## References

[B1] Blackwelder RE (1936) Morphology of the coleopterous family Staphylinidae. Smithsonian Miscellaneous Collections 94 (13): 1-102.

[B2] Briceño RD Eberhard WG (1995) The functional morphology of male cerci and associated characters in 13 species of tropical earwigs (Dermaptera: Forficulidae, Labiidae, Carcinophoridae, Pygidicranidae). Smithsonian Contributions to Zoology 555: 1-63. doi: 10.5479/si.00810282.555

[B3] Brindle A (1987) Order Dermaptera. In: Stehr FW (Ed) Immature Insects [Volume 1]. Kendall Hunt Publishing, Dubuque, IA, 171–178 [total volume xiv+754 pp.]

[B4] Chatzimanolis S Engel MS (2010) *Laasbium* Scudder: A genus of Tertiary earwigs, not rove beetles, and the classification of Florissant fossil Dermaptera (Insecta). Annales Zoologici 60 (1): 101-108. doi: 10.3161/000345410X499588

[B5] Cockerell TDA (1920) Fossil arthropods in the British Museum – IV. Annals and Magazine of Natural History, Series 9 6: 211–214.

[B6] Costa JT (2006) The Other Insect Societies. Harvard University Press, Cambridge, MA, xiv+[i]+767 pp.

[B7] Cruickshank RD Ko K (2003) Geology of an amber locality in the Hukawng Valley, northern Myanmar. Journal of Asian Earth Sciences 21 (5): 441-455. doi: 10.1016/S1367-9120(02)00044-5

[B8] Davies RG (1966) The postembryonic development of *Hemimerus vicinus* Rehn & Rehn (Dermaptera: Hemimeridae). Proceedings of the Royal Entomological Society of London, Series A, General Entomology 41 (4–6):67-77.

[B9] Engel MS (2001) A monograph of the Baltic amber bees and evolution of the Apoidea (Hymenoptera). Bulletin of the American Museum of Natural History 259: 1-192. doi: 10.1206/XXXXXX

[B10] Engel MS (2009) Gregarious behaviour in Cretaceous earwig nymphs (Insecta, Dermaptera) from southwestern France. Geodiversitas 31 (1): 129-135. doi: 10.5252/g2009n1a11

[B11] Engel MS (2011) Systematic melittology: Where to from here? Systematic Entomology 36(1): 2–15. doi: 10.1111/j.1365-3113.2010.00544.x

[B12] Engel MS Chatzimanolis S (2005) Early Cretaceous earwigs (Dermaptera) from the Santana Formation, Brazil. Polskie Pismo Entomologiczne 74 (3): 219-226.

[B13] Engel MS Haas F (2007) Family-group names for earwigs (Dermaptera). American Museum Novitates 3567: 1-20. doi: 10.1206/0003-0082(2007)539[1:FNFED]2.0.CO;2

[B14] Engel MS Grimaldi DA (2004) A primitive earwig in Cretaceous amber from Myanmar (Dermaptera: Pygidicranidae). Journal of Paleontology 78 (5): 1018-1023. doi: 10.1666/0022-3360(2004)078<1018:APEICA>2.0.CO;2

[B15] Engel MS Grimaldi DA Krishna K (2007) Primitive termites from the Early Cretaceous of Asia (Isoptera). Stuttgarter Beiträge zur Naturkunde, Serie B, Geologie und Paläontologie 371: 1-32.

[B16] Engel MS Grimaldi DA Krishna K (2009) Termites (Isoptera): Their phylogeny, classification, and rise to ecological dominance. American Museum Novitates 3650: 1-27. doi: 10.1206/651.1

[B17] Engel MS Lim J-D Baek K-S Martin LD (2002) An earwig from the Lower Cretaceous of Korea (Dermaptera: Forficulina). Journal of the Kansas Entomological Society 75 (2): 86-90.

[B18] Engel MS Ortega-Blanco J Azar D (2011) The earliest earwigs in amber (Dermaptera): A new genus and species from the Early Cretaceous of Lebanon. Insect Systematics and Evolution 42 (2): 139-148. doi: 10.1163/187631211X555717

[B19] Giles ET (1961) Further studies on the growth stages of *Arixenia esau* Jordan and *Arixenia jacobsoni* Burr (Dermaptera: Arixeniidae), with a note on the first instar antennae of *Hemimerus talpoides* Walker (Dermaptera: Hemimeridae). Proceedings of the Royal Entomological Society of London, Series A, General Entomology 36 (1–3):21-26.

[B20] Giles ET (1963) The comparative external morphology and affinities of the Dermaptera. Transactions of the Royal Entomological Society of London 115 (4): 95-164. doi: 10.1111/j.1365-2311.1963.tb00816.x

[B21] Green EE (1896) Notes on *Dyscritina longisetosa* Westw. Transactions of the Royal Entomological Society of London 1896: 229-231.

[B22] Green EE (1898) Further notes on *Dyscritina* Westw. Transactions of the Royal Entomological Society of London 1898: 381–387, +2pls [pls. 18, 19].

[B23] Grimaldi D Agosti D (2000) A formicine in New Jersey Cretaceous amber (Hymenoptera: Formicidae) and early evolution of the ants. Proceedings of the National Academy of Sciences, USA 97 (25): 13678-13683. doi: 10.1073/pnas.240452097 PMC17635 11078527

[B24] Grimaldi D Engel MS (2005) Evolution of the Insects. Cambridge University Press, Cambridge, xv+755 pp.

[B25] Grimaldi D Agosti D Carpenter JM (1997) New and rediscovered primitive ants (Hymenoptera: Formicidae) in Cretaceous amber from New Jersey, and their phylogenetic relationships. American Museum Novitates 3208: 1-43.

[B26] Grimaldi DA Engel MS Nascimbene PC (2002) Fossiliferous Cretaceous amber from Myanmar (Burma): Its rediscovery, biotic diversity, and paleontological significance. American Museum Novitates 3361: 1-72. doi: 10.1206/0003-0082(2002)361<0001:FCAFMB>2.0.CO;2

[B27] Grimaldi DA Engel MS Krishna K (2008) The species of Isoptera (Insecta) from the Early Cretaceous Crato Formation: A revision. American Museum Novitates 3626: 1-30. doi: 10.1206/616.1

[B28] Günther K Herter K (1974) Dermaptera (Ohrwürmer). Handbuch der Zoologie: Eine Naturgeschichte der Stämme des Tierreiches. IV Band: Arthropoda – 2 Hälfte: Insecta, Zweite Auflage, 2 Teil: Spezielles 11: 1–158.

[B29] Haas F (1995) The phylogeny of the Forficulina, a suborder of the Dermaptera. Systematic Entomology 20 (2): 85-98. doi: 10.1111/j.1365-3113.1995.tb00085.x

[B30] Haas F (2003) Ordnung Dermaptera, Ohrwürmer. In: Dathe HH (Ed) Lehrbuch der Speziellen Zoologie. Band I: Wirbellose Tiere. 5 Teil: Insecta. Spektrum Akademischer Verlag, Heidelberg, Germany, 173–180 [total volume xii+[i]+961 pp.]

[B31] Haas F (2007) Dermaptera: Earwigs. In: Martill DM, Bechly G, Loveridge RF (Eds) The Crato Fossil Beds of Brazil: Window into an Ancient World. Cambridge University Press, Cambridge, 222–234 [total volume xvi+625 pp.]

[B32] Haas F Klass K-D (2003) The basal phylogenetic relationships in the Dermaptera. Entomologische Abhandlungen 61 (2): 138-142.

[B33] Hand S Archer M Bickel D Creaser P Dettmann M Godthelp H Jones A Norris B Wicks D (2010) Australian Cape York amber. In: Penney D (Ed) Biodiversity of Fossils in Amber from the Major World Deposits. Siri Scientific Press, Manchester, 69–79 [total volume 304 pp.]

[B34] Hincks WD (1955) A Systematic Monograph of the Dermaptera of the World Based on Material in the British Museum (Natural History). Part One. Pygidicranidae subfamily Diplatyinae. British Museum (Natural History), London, ix+132 pp.

[B35] Kamp T van de Greven H (2010) On the architecture of beetle elytra. Entomologie Heute 22: 191-204.

[B36] Klass K-D (2001) The female abdomen of the viviparous earwig *Hemimerus vosseleri* (Insecta: Dermaptera: Hemimeridae), with a discussion of the postgenital abdomen of Insecta. Zoological Journal of the Linnean Society 131 (3): 251-307. doi: 10.1111/j.1096-3642.2001.tb02239.x

[B37] Matzke D Klass K-D (2005) Reproductive biology and nymphal development in the basal earwig *Tagalina papua* (Insecta: Dermaptera: Pygidicranidae), with a comparison of brood care in Dermaptera and Embioptera. Entomologische Abhandlungen 62 (2): 99-116.

[B38] McKellar RC Wolfe AP (2010) Canadian amber. In: Penney D (Ed) Biodiversity of Fossils in Amber from the Major World Deposits. Siri Scientific Press, Manchester, 149–166 [total volume 304 pp.].

[B39] Nakata S Maa TC (1974) A review of the parasitic earwigs (Dermaptera: Arixeniina; Hemimerina). Pacific Insects 16 (2–3):307-374.

[B40] Naomi S-I (1988) Comparative morphology of the Staphylinidae and the allied groups (Coleoptera, Staphylinoidea). V. Cervix and prothorax. Kontyû 56 (3): 506-513.

[B41] Naomi S-I (1989) Comparative morphology of the Staphylinidae and the allied groups (Coleoptera, Staphylinoidea). IX. General structure, lateral plates, stigmata and 1st to 7th segments of abdomen. Japanese Journal of Entomology 57 (3): 517-526.

[B42] Nel A Waller A Albouy V Menier J-J De Ploëg G (2003) New fossil earwigs from the lowermost Eocene amber of Paris Basin (France) (Insecta, Dermaptera, family incertae sedis). Geodiversitas 25 (1): 119-129.

[B43] Peñalver E Delclòs X (2010) Spanish amber. In: Penney D (Ed) Biodiversity of Fossils in Amber from the Major World Deposits. Siri Scientific Press, Manchester, 236–270 [total volume 304 pp.]

[B44] Perrichot V Lacau S Néraudeau D Nel A (2008a) Fossil evidence for the early ant evolution. Naturwissenschaften 95 (2): 85-90. doi: 10.1007/s00114-007-0301-8 17891532

[B45] Perrichot V Nel A Néraudeau D Lacau S Guyot T (2008b) New fossil ants in French Cretaceous amber (Hymenoptera: Formicidae). Naturwissenschaften 95 (2): 91-97. doi: 10.1007/s00114-007-0302-7 17828384

[B46] Perrichot V Engel MS Nel A Tafforeau P Soriano C (2011) New earwig nymphs (Dermaptera: Pygidicranidae) in mid-Cretaceous amber from France. Cretaceous Research 32 (3): 325-330. doi: 10.1016/j.cretres.2011.01.004

[B47] Popham EJ (1959) The anatomy in relation to feeding habits of *Forficula auricularia* L. and other Dermaptera. Proceedings of the Zoological Society of London 133 (2): 251-300. doi: 10.1111/j.1469-7998.1959.tb05563.x

[B48] Popham EJ (1965) A key to dermapteran subfamilies. Entomologist 98: 126-136.

[B49] Popham EJ (1985) The mutual affinities of the major earwig taxa (Insecta, Dermaptera). Zeitschrift für Zoologische Systematik und Evolutionforschung 23 (3): 199-214.

[B50] Rankin SM Palmer JO (2009) Dermaptera (earwigs). In: Resh VH, Cardé RT (Eds) Encyclopedia of Insects [2nd Edition]. Academic Press, San Diego, CA, 259–261 [total volume xxxiii+[iii]+1132 pp.]

[B51] Ross AJ Mellish C York P Crighton B (2010) Burmese amber. In: Penney D (Ed) Biodiversity of Fossils in Amber from the Major World Deposits. Siri Scientific Press, Manchester, 208–235 [total volume 304 pp.]

[B52] Rust J Singh H Rana RS McCann T Singh L Anderson K Sarkar N Nascimbene PC Stebner F Thomas JC Solórzano-Kraemer M Williams CJ Engel MS Sahni A Grimaldi D (2010) Biogeographic and evolutionary implications of a diverse paleobiota in amber from the Early Eocene of India. Proceedings of the National Academy of Sciences, USA 107 (43): 18360-18365. 10.1073/pnas.1007407107 PMC2972964 20974929

[B53] Solórzano-Kraemer MM (2007) Systematic, palaeoecology, and palaeobiogeography of the insect fauna from Mexican amber. Palaeontographica, Abteilung A: Paläozoologie – Stratigraphie 282: 1–133, +14 pls.

[B54] Solórzano-Kraemer MM (2010) Mexican amber. In: Penney D (Ed) Biodiversity of Fossils in Amber from the Major World Deposits. Siri Scientific Press, Manchester, 42–56 [total volume 304 pp.]

[B55] Steinmann H (1986) Dermaptera: Catadermaptera I. Das Tierreich: Eine Zusammenstellund und Kennzeichnung der rezenten Tierformen 102: xiv+1–343.

[B56] Waller A Caussanel C Jamet C (1996) Variation morphologique des pièces buccales chez quelques Dermaptères. Bulletin de la Société Entomologique de France 101 (5): 523-533.

[B57] Waller A Caussanel C Jamet C Albouy V (1999) Etude comparée des pièces thoraciques et de leurs appendices chez quelques Dermaptères. Bulletin de la Société Entomologique de France 104 (5): 427-440.

[B58] Wappler T Engel MS Haas F (2005) The earwigs (Dermaptera: Forficulidae) from the middle Eocene Eckfeld maar, Germany. Polskie Pismo Entomologiczne 74 (3): 227-250.

[B59] Willmann R (1990) Die Bedeutung paläontologischer Daten für die zoologische Systematik. Verhandlungen der Deutschen Zoologischen Gesellschaft 83: 277-289.

[B60] Zhang H-C (1997) Early Cretaceous insects from the Dalazi Formation of the Zhixin Basin, Jilin Province, China. Palaeoworld 7: 75-103.

[B61] Zhang J-F (2002) The most primitive earwigs (Archidermaptera, Dermaptera, Insecta) from the Upper Jurassic of Nei Monggol Autonomous Region, northeastern China. Acta Micropalaeontologica Sinica 19 (4): 348-362.

[B62] Zhao J-X Ren D Shih C-K (2010a) Enigmatic earwig-like fossils from Inner Mongolia, China. Insect Science 17 (5): 459-464.

[B63] Zhao J-X Zhao Y-Y Shih C-K Ren D Wang Y-J (2010b) Transitional fossil earwigs – a missing link in Dermaptera evolution. BMC Evolutionary Biology 10: 344 [1–10] doi: 10.1186/1471-2148-10-344 PMC2993717 21062504

[B64] Zhao J-X Shih C-K Ren D Zhao Y-Y (2011) New primitive fossil earwig from Daohugou, Inner Mongolia, China (Insecta: Dermaptera: Archidermaptera). Acta Geologica Sinica 85 (1): 75-80. doi: 10.1111/j.1755-6724.2011.00380.x

[B65] Zherikhin VV Ross AJ (2000) A review of the history, geology and age of Burmese amber (burmite). Bulletin of the Natural History Museum, London (Geology) 56 (1): 3-10.

